# A pilot study on the effect of advance care planning implementation on healthcare utilisation and satisfaction in patients with advanced heart failure

**DOI:** 10.1007/s12471-022-01705-8

**Published:** 2022-06-21

**Authors:** J. E. Coster, G. H. ter Maat, M. L. Pentinga, A. K. L. Reyners, D. J. van Veldhuisen, P. de Graeff

**Affiliations:** 1 Department of Cardiology, University Medical Centre Groningen, University of Groningen, Groningen, The Netherlands; 2 Department of Internal Medicine, University Medical Centre Groningen, University of Groningen, Groningen, The Netherlands; 3 Department of Cardiology, Wilhelmina Hospital Assen, Assen, The Netherlands; 4 Department of Medical Oncology, University Medical Centre Groningen, University of Groningen, 9700 RB Groningen, The Netherlands; 5 Department of Internal Medicine, University Centre of Geriatric Medicine, University Medical Centre Groningen, University of Groningen, Groningen, The Netherlands

**Keywords:** Advance care planning, Heart failure, Palliative care, Stakeholder participation, Continuity of patient care

## Abstract

**Background:**

Patients with advanced heart failure may benefit from palliative care, including advance care planning (ACP). ACP, which can include referral back to the general practitioner (GP), may prevent unbeneficial hospital admissions and interventional/surgical procedures that are not in accordance with the patient’s personal goals of care.

**Aim:**

To implement ACP in patients with advanced heart failure and explore the effect of ACP on healthcare utilisation as well as the satisfaction of patients and cardiologists.

**Methods:**

In this pilot study, we enrolled 30 patients with New York Heart Association class III/IV heart failure who had had at least one unplanned hospital admission in the previous year because of heart failure. A structured ACP conversation was held and documented by the treating physician. Primary outcome was the number of visits to the emergency department and/or admissions within 3 months after the ACP conversation. Secondary endpoints were the satisfaction of patients and cardiologists as established by using a five-point Likert scale.

**Results:**

Median age of the patients was 81 years (range 33–94). Twenty-seven ACP documents could be analysed (90%). Twenty-one patients (78%) did not want to be readmitted to the hospital and subsequently none of them were readmitted during follow-up. Twenty-two patients (81%) discontinued all hospital care. All patients who died during follow-up (*n* = 12, 40%) died at home. Most patients and cardiologists indicated that they would recommend the intervention to others (80% and 92% respectively).

**Conclusion:**

ACP, and subsequent out-of-hospital care by the GP, was shown to be applicable in the present study of patients with advanced heart failure and evident palliative care needs. Patients and cardiologists were satisfied with this intervention.

## What’s new?

- In our cohort of 30 selected patients with advanced heart failure a structured advance care planning (ACP) conversation was held by the cardiologist.
- The majority of the patients did not want to be readmitted to the hospital and were successfully treated at home by their general practitioner.
- No unwanted hospital readmissions occurred during the 3‑month follow-up.
- The majority of our patients were alive at the end of the 3‑month follow-up, emphasising the need for early ACP to facilitate treatment in accordance with their personal goals of care.
- Both patients and physicians were satisfied with the intervention, as they felt explicit communication on prognosis prevented mutual misunderstanding.

## Introduction

Despite therapeutic advances and ongoing research, advanced heart failure remains associated with frequent hospital admissions, poor prognosis and high costs [1–3]. As patients with advanced heart failure often suffer pronounced functional decline and poor quality of life, they could benefit from palliative care [4]. The need for palliative care in heart failure is increasingly recognised [5, 6], yet it is far from being widely implemented [2, 7]. There are several barriers to its implementation, such as the unpredictable disease trajectory of chronic heart failure and the misconception that palliative care is restricted to end-of-life care [8].

An important aspect of palliative care is advance care planning (ACP). ACP is defined as the ability to enable individuals to define goals and preferences for future medical treatment and care, to discuss these goals and preferences with family and healthcare providers, and to record and review these preferences if appropriate [9]. ACP in patients with heart failure may include symptom management, preferences for treatment options, including surgical or catheter procedures, management of cardiac devices, end-of-life preferences and advance directives [10]. ACP in a general elderly population has been associated with increased patient satisfaction, a higher concordance between patients’ wishes and the treatment received and an increased chance of dying at home [11]. ACP may cover end-of-life preferences but can and should be applied earlier in the disease trajectory to maximise its effect.

Early application of ACP in heart failure may also lead to a reduction in healthcare expenses and less utilisation of acute medical care [12, 13]. Timely discussion of the patients’ goals of care may prevent futile yet expensive hospital admissions, especially in the last 3 months of life. For some patients it may be in accordance with their treatment goals to be fully discharged from hospital care (including outpatient visits) to the care of their general practitioner (GP).

Despite the general belief that ACP could improve quality of life for patients with advanced heart failure, experience with this strategy is scarce in the Netherlands. To gain experience we performed a pilot study to implement ACP in a single cohort of selected patients with advanced heart failure. The primary objective of this pilot study was to assess healthcare utilisation, defined as the number of visits to the emergency department and hospital admissions. In addition, the satisfaction of patients and cardiologists with ACP conversations was investigated and data on 3‑month all-cause mortality were obtained.

## Methods

### Study design and participants

The present investigation was a pilot study to implement ACP in a single cohort of patients with advanced heart failure. To include a representative sample this study was conducted in a university hospital (University Medical Centre Groningen) with facilities for advanced heart failure care, including transplant and mechanical circulatory support, as well as in a district hospital (Wilhelmina Hospital Assen) in the Netherlands.

Hospitalised patients with advanced heart failure (New York Heart Association (NYHA) class III/IV) were enrolled if they met the following inclusion criteria: (1) one or more unplanned hospital admissions related to heart failure within the previous year or (2) limited treatment options due to comorbidity and/or a limited life expectancy as assessed by the attending cardiologist. By using these inclusion criteria, we aimed to select a population with evident palliative care needs, making the threshold for performing ACP as low as possible.

The study was conducted in accordance with the Declaration of Helsinki and Good Clinical Practice guidelines. Permission was granted by the local medical ethics committee. All patients gave their informed consent.

### Intervention

The intervention consisted of an ACP conversation with patients and their caregivers. ACP conversations were held and documented by the treating cardiologist and/or cardiology resident. The implementation of ACP was coordinated by a cardiologist who participates in the palliative care team of the university hospital and received additional training in palliative care (J.C.). A specialised palliative care physician participated upon request. Family members were encouraged to be present during ACP conversations. All ACP conversations were held in consultation with the patients’ GP. The issues that were addressed are summarised in Tab. 1.

**Table 1:** Themes discussed in advance care planning conversation

Theme	
Symptoms and complaints	Current and anticipated symptoms with their treatment options All domains of palliative care (physical, psychological, social, spiritual)
Heart failure treatment	Preferred place of care (at home, by GP, at outpatient clinic) Wishes regarding hospital readmission or management at home
Advance directives	DNR, invasive ventilation, surgical procedures (either diagnostic or therapeutic) Management of devices, such as turning off shock function of ICD
End-of-life care	Preferred place of death Previously drawn-up living will Exploratory conversation on palliative sedation and euthanasia

*GP* general practitioner, *DNR* do-not-resuscitate order, *ICD* implantable cardioverter defibrillator

Topics discussed were documented in an ACP document [14]. Upon hospital discharge, this document was added to the electronic medical record, was sent to the GP and other involved healthcare providers, and the patient received a copy.

### Outcome measures

The primary outcome was the number of visits to the emergency department and/or hospital admissions within 3 months following hospital discharge, as documented in the medical records.

Secondary endpoints were the satisfaction of patients and physicians and 3‑month all-cause mortality. Satisfaction was assessed 1 week after the intervention using a short questionnaire in which the patient and physician were asked to rate one statement on a five-point Likert scale ranging from 1 (strongly disagree) to 5 (strongly agree). In both questionnaires respondents were asked to explain their answers.

Demographic patient variables were obtained from the medical records.

### Statistical analysis

All data were documented using descriptive statistics. Data analysis was performed using the software package IBM SPSS Statistics, version 23.0 (SPSS, Inc., Chicago, IL, USA).

## Results

### Patients

Between September 2017 and August 2019, 30 patients were enrolled in the study (Tab. 2). Twenty-eight patients (93%) were enrolled at the university hospital. Median age was 81 years (range 33-94 years); most patients were female (63%). The most frequently reported comorbidities were diabetes (40%), chronic pulmonary disease (30%) and severe renal impairment (23%), as defined by the Charlson Comorbidity Index [15].

**Table 2:** Baseline characteristics

	*n*	%
*Age, years (median, range)*	81 (33–94)	
*Sex*		
Male	11	37%
Female	19	63%
*Number of hospital admissions in past year*		
0	7	23%
1	11	37%
≥ 2	12	40%
*Charlson Comorbidity Index*		
1	8	27%
2	4	13%
3	7	23%
≥ 4	11	37%
*Heart failure type*		
HFrEF	16	53%
HFpEF	14	47%
*NYHA classification*		
Class III	25	83%
Class IV	5	17%
*NTproBNP levels on admission in ng/l (median, range)*		
HFrEF	13,767	
HFpEF	5084	
*Device*		
None	21	70%
Pacemaker	4	13%
ICD	2	7%
CRT‑D	2	7%
Unknown	1	3%
*LVEF, % (median, range)*		
HFrEF	31 (15–40)	
HFpEF	55 (45–60)	
*Duration of heart disease prior to inclusion, years (median, range)*	2 (0–34)	
*Aetiology of HFrEF (n* *=* *16)*		
Ischaemic heart disease	8	50%
Cardiomyopathy	2	12%
Unknown	6	38%
*Medication use on admission in HFrEF patients (n* *=* *16)*		
ACE inhibitor or ARB	8	50%
Beta blocker	9	56%
MRA	7	44%
All of the above	5	31%
Data on medication missing	2	13%

*HFrEF* heart failure with reduced ejection fraction, *HFpEF* heart failure with preserved ejection fraction, *NYHA* New York Heart Association, *NTproBNP* N-terminal pro-brain natriuretic peptide, *ICD* internal cardiac defibrillator, *CRT‑D* cardiac resynchronisation therapy with defibrillator, *BMI* body mass index, *LVEF* left ventricular ejection fraction, *ACE* angiotensin-converting enzyme, *ARB* angiotensin II receptor blocker, *MRA* mineralocorticoid receptor antagonist

Sixteen patients (53%) suffered from heart failure with reduced ejection fraction (HFrEF). All patients had severe symptoms of heart failure (83% NYHA III and 17% NYHA IV) and markedly elevated levels of N‑terminal pro-brain natriuretic peptide.

### ACP documentation

Twenty-seven ACP documents were available for analysis (Tab. 3). In three patients (10%), the ACP document was not saved in the medical record and could thus not be used for this study. In 10 patients (37%) the palliative care team was consulted. Reasons for consultation of the palliative care team included the presence of complex problems in multiple palliative care domains and aid in ACP conversations.

**Table 3:** Contents of advance care planning (*ACP*) documentation

	*n*	%
*Number of ACP documents available for analysis*	27	90%
*Advance directives*		
Do-not-resuscitate order		
– Yes	26	96%
– No	1	4%
– Unknown	0	0%
Wish to be readmitted to hospital		
– Yes	5	18%
– No	21	78%
– Unknown/not discussed	1	4%
Preferred place of death		
– Home	20	74%
– Hospice	1	4%
– Not discussed	6	22%
*Number of patients with defined problem in palliative domain*		
– Physical domain	25	93%
– Psychological domain	22	81%
– Social domain	21	78%
– Spiritual domain	17	63%

Advance directives were documented in all 27 cases and contained do-not-resuscitate orders in almost all of the patients (*n* = 26, 96%). All patients with an implantable cardioverter defibrillator (ICD) decided to deactivate the shock function. In 24 patients (89%) it was documented whether palliative sedation and euthanasia had been discussed.

### Medication

Medication data were complete for 14 HFrEF patients (88%, Tab. 2). The majority of these patients (69%) did not tolerate full heart failure therapy prior to admission due to the severity of their underlying heart disease and/or comorbidity.

After ACP, five HFrEF patients (35%) wished to continue all disease-modifying heart failure therapy. Five other patients (35%) were discharged with a medication regime fitted to their symptoms, i.e. discontinuation of angiotensin-converting enzyme inhibitor because of symptomatic hypotension. Four patients (28%) had no wish for further treatment or a short estimated life expectancy and were discharged without any heart failure medication or only for symptomatic relief.

As there is currently no evidence-based treatment for heart failure with preserved ejection fraction, treatment was tailor-made to patients’ symptoms and goals.

### Follow-up

Three patients presented at the emergency department and were readmitted within 3 months after ACP documentation. In two patients, it had been documented that they wished to be readmitted based on personal preferences. For one patient, it was not documented whether readmission had been discussed.

After consulting with the patient and their GP, 22 patients (81%) were discharged from hospital care, including outpatient visits. Most patients (78%) did not want to be readmitted and preferred to die at home. None of these patients were readmitted during follow-up, nor did they undergo invasive diagnostic procedures.

**Fig. 1:**
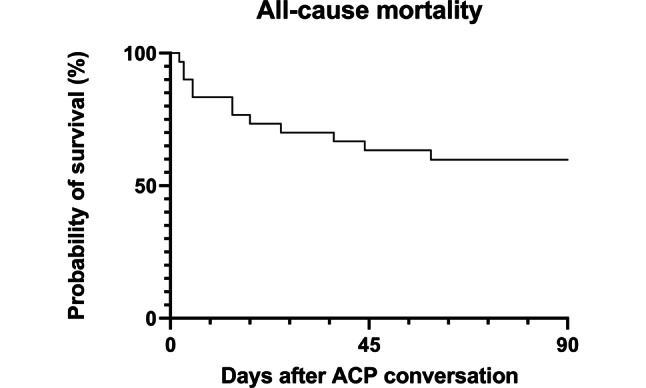
Kaplan-Meier curve representing patient survival after the advance care planning intervention

The treating cardiologist assessed life expectancy to be < 2 weeks in two patients (7%), < 3 months in four patients (15%), < 1 year in six patients (22%) and unclear in 15 patients (56%). Even though only two patients had an estimated life expectancy of less than 2 weeks, seven patients (23%) died within 2 weeks after discharge. Three-month all-cause mortality was 40% (*n* = 12) (Fig. 1). All patients died either at home or in a hospice, in agreement with their wishes.

### Satisfaction of patients and physicians

Satisfaction with the intervention could be evaluated in 10 patients (33%). The other 20 patients passed away or could not be reached for follow-up. Eight patients (80%) were satisfied or very satisfied and would recommend this intervention to other patients. Two patients were neutral, of whom one mentioned that the ACP conversation led to anxiety and distress.

The satisfaction of treating physicians could be evaluated in 28 of 30 cases (93%). The majority stated they were likely or very likely to recommend implementation of ACP in daily practice (87%).

Qualitative analysis of the written questionnaire showed that the intervention encouraged physicians to be more explicit about life expectancy. The clarity provided made it easier to discuss the patient’s wishes for the last phase of life. By explicitly identifying patients with palliative care needs, cardiologists hoped to help other caregivers to respond appropriately to any problems that might occur, thereby preventing unwanted hospital admissions.

## Discussion

The results of our study suggest that ACP for patients with advanced heart failure is successful and satisfactory for both patients and their physicians. In almost all patients the intervention led to documentation of advance directives such as a do-not-resuscitate order and not to undergo invasive diagnostic procedures or treatments. The majority of the patients did not want to be readmitted and were treated in their home environment in accordance with their wishes.

### Timing of ACP in advanced heart failure

Treatment guidelines generally mention palliative care when describing end-of-life care [6, 16], although the need for early palliative care and ACP as an integrated part of comprehensive heart failure care is increasingly recognised [5, 17, 18]. The persistent misunderstanding that palliative care and ACP are only applicable for patients at the end of life [19] may hamper early palliative care implementation. For example, in our cohort almost a quarter of the patients died in the first 2 weeks of follow-up. When to start ACP conversations with heart failure patients has yet to be determined, whereas the palliative care field encourages early integration of palliative care in disease-modifying treatment [20]. Although many physicians find it difficult to find the right moment for discussing ACP [19], we advocate early ACP starting at diagnosis, especially in elderly patients [21]. Instead of end-of-life care, early ACP may focus on values regarding quality of life and goals of care.

### Benefits of ACP

The majority of our study population did not want to be readmitted to the hospital and could be satisfactorily cared for at home. Well-timed documentation of the patients’ treatment goals enables professional caregivers to treat the patient according to their wishes and prevents unwanted hospital admissions [22, 23], thereby improving patient-centred outcomes [24] and reducing healthcare utilisation and costs [25].

Applying ACP earlier in the treatment process may also be beneficial in avoiding invasive procedures, which are costly and from which this patient category will scarcely benefit. An example of timely ACP is discussing whether replacement of an ICD battery is in line with the patient’s goals and future perspectives as heart failure and other comorbidities progresses over time. All ICD patients in our study wished to deactivate the shock function after an ACP conversation. In an outpatient setting the possibility of deactivation of an ICD should ideally be addressed early, perhaps already at the time of implantation [26]. Certainly, no decision has to be made at that time, yet providing early information makes the subject accessible, may prevent misunderstandings and could facilitate shared decision making [27].

### Strengths and limitations

A strength of our study is the fact that most of the ACP was done by the cardiologists (in training) themselves. The palliative care team was involved in only 37% of patients. The satisfaction of cardiologists conducting ACP has scarcely been reported to date; our results are a first draft for further research on this topic.

Limitations of this study are the small number of patients, lack of a control group and non-randomised design. A more complex study design was considered, but as the cardiologists were unfamiliar with ACP, a known reason for not applying ACP [28], we feared that the required number of participants would not be achieved. Recruiting participants for the present study proved to be laborious as well. However, during the course of the study, the implementation of the intervention seemed to contribute to a starting mind shift in our staff, which led to the threshold for ACP being lowered. Another limitation is the fact that patient satisfaction could be assessed in only a subgroup of patients, which is partly a consequence of the high mortality in the weeks following ACP. In addition, patient satisfaction was not followed over time, especially in patients with a longer survival. Follow-up of patient satisfaction and other endpoints relevant to ACP, such as quality of life, would be an interesting subject for future studies.

## Conclusion

In conclusion, our results suggest that implementing ACP in patients with heart failure may lead to tailor-made care, in which the majority of the patients could be successfully referred to the GP with mutual satisfaction. Making ACP a part of regular heart failure care may lead to improved quality-of-life outcomes and prevent unwanted hospital admissions and invasive treatments.
